# Hybrid Cardiac Rehabilitation as an Optimal Strategy for Post-MI Recovery: A 14-Week Prospective Study on Clinical and Functional Outcomes

**DOI:** 10.3390/healthcare14091231

**Published:** 2026-05-03

**Authors:** Liviu Ionuț Șerbănoiu, Stefan Sebastian Busnatu, Dragos Trache, Gabriel Olteanu, Elena Serbanoiu, Abdul Basit, Narcisa Busnatu, Mihaela Mandu, Gelu Onose, Francesco Perone, Florin Mitu, Cătălina Liliana Andrei, Crina Julieta Sinescu

**Affiliations:** 1 Department of Cardio-Thoracic Pathology, Faculty of Medicine, Carol Davila University of Medicine and Pharmacy, 050474 Bucharest, Romania; dragos-alin.trache@rez.umfcd.ro (D.T.); gabriel.olteanu@stud.umfcd.ro (G.O.); elena.plesu@rez.umfcd.ro (E.S.); narcisaalazar@gmail.com (N.B.); mihaela.mandu@umfcd.ro (M.M.); gelu.onose@umfcd.ro (G.O.); ccatalina97@yahoo.com (C.L.A.); crina.sinescu@umfcd.ro (C.J.S.); 2 Department of Cardiology, Emergency Hospital Bagdasar-Arseni, 050474 Bucharest, Romania; 3 Department of Internal Medicine, Liaquat National Hospital and Medical College, Karachi 74800, Pakistan; ssangah@yahoo.com; 4 Physical and Rehabilitation Medicine Department, Emergency Hospital Bagdasar-Arseni, 050474 Bucharest, Romania; 5 Cardiac Rehabilitation Unit, Rehabilitation Clinic ‘Villa delle Magnolie’, Castel Morrone, 81020 Caserta, Italy; francescoperone1988@gmail.com; 6 Faculty of Medicine, “Grigore T. Popa” University of Medicine and Pharmacy, 700115 Iasi, Romania; mitu.florin@yahoo.com; 7 Department of Cardiology, Clinical Rehabilitation Hospital, 700661 Iasi, Romania

**Keywords:** hybrid cardiac rehabilitation, myocardial infarction, NYHA class II heart failure, exercise capacity, wearable-enabled remote monitoring, body composition

## Abstract

**Background**: Hybrid cardiac rehabilitation (CR) combining supervised and home-based phases with wearable monitoring may improve access and outcomes after myocardial infarction (MI). Objective: To assess the impact of a 14-week hybrid CR program on functional class, exercise capacity, hemodynamics, body composition, and physical activity in post-MI patients with NYHA class II symptoms. **Methods**: Sixty-six adults post-MI underwent 2 weeks of in-hospital initiation followed by 12 weeks of home-based rehabilitation via a smartwatch–smartphone platform. Within-subject changes from baseline to week 14 were analyzed using appropriate paired statistical tests. **Results**: NYHA class improved significantly, with 39% of participants downgrading their class (*p* < 0.001). Body weight decreased by 1.27 ± 2.51 kg (*p* < 0.001). Systolic and diastolic blood pressures declined (both *p* ≤ 0.002). Maximal METS rose markedly (25.7% increase; *p* < 0.001), and watts/kg improved (*p* < 0.001). Resting heart rate decreased (*p* = 0.002); peak exercise heart rate change was non-significant. Fat mass declined and skeletal muscle mass increased (mean gain 0.98 kg; *p* < 0.001). Daily step count increased from 5550 ± 2026 to 7267 ± 2500 steps (*p* < 0.001). Total body water also increased (*p* < 0.001). **Conclusions**: The hybrid CR program produced significant improvements in functional class, exercise capacity, blood pressure, body composition, and physical activity in post-MI NYHA II patients, supporting its effectiveness as a remotely enabled secondary prevention strategy. However, the results are based on hypotheses and randomized controlled trials must confirm the benefits especially with a control group. Nonetheless, it is a feasible and potentially effective alternative to conventional programs in resource-limited settings.

## 1. Introduction

Cardiovascular diseases (CVDs) remain the leading cause of mortality worldwide. According to recent global estimates, CVD accounts for about 17.9 million deaths per year (roughly 31% of all deaths) [1]. A major contributor is coronary artery disease; more than 7 million people suffer an acute myocardial infarction (MI) each year globally [2]. Despite advances in acute cardiac care, many MI survivors face high risks. Approximately 10% will die within one year and 20% experience a new cardiovascular event in the first year [2]. Moreover, a significant proportion develop left ventricular dysfunction or heart failure and are often New York Heart Association (NYHA) class II following MI, which further elevates morbidity. This global disease burden underlines the critical need for effective secondary prevention strategies such as cardiac rehabilitation (CR).

Cardiac rehabilitation (CR) is a tailored, evidence-based, and monitored intensive regimen that combines exercise training, health education, physical activity promotion, and detailed counseling to control cardiovascular risk factors [3]. Participation in CR after MI or by heart failure patients is strongly recommended by clinical guidelines (Class I, Level A), the American Heart Association/American College of Cardiology (AHA/ACC) and the European Society of Cardiology (ESC), due to its well-demonstrated benefits [4]. Exercise-based CR can improve functional capacity, enhance quality of life, and reduce recurrent hospitalizations. Meta-analyses have shown that CR confers a significant survival benefit such as reducing cardiovascular mortality by approximately 20 to 25% compared to usual care [5]. These benefits apply across various cardiac populations, including post-MI patients and those with heart failure with reduced ejection fraction [6]. CR is therefore a cornerstone of post-MI care and heart failure management, aiming to restore patients’ functional status and prevent further cardiac events.

Despite its proven efficacy, CR remains grossly under-utilized worldwide. In practice, only a minority of eligible patients enroll in or complete rehabilitation programs [7,8]. A recent systematic review reported that the average CR participation rate after acute MI is only approximately 34% [2]. Furthermore, a 2023 trial protocol reports that although the benefits of CR are emphasized in guidelines, actual participation rates are low worldwide; only 24.4% of Medicare beneficiaries took part in CR, only 26.9% completed it, and global participation remains about 30–40% [9]. Participation is even lower in many low-resource settings and among certain high-risk groups (such as older patients or those with multiple comorbidities). One major barrier is the limited availability of CR programs globally; only about 38.8% of countries have any cardiac rehabilitation services at all [5]. Even in countries with CR, access is constrained by factors like geographic distance, cost, inadequate referral, and capacity limits, with an estimated density of as low as one program per several million inhabitants in low-income regions [5]. These gaps lead to many post-MI and heart failure patients missing out on CR’s life-saving benefits. There is a clear imperative to explore innovative delivery models to expand CR access and adherence on a global scale [4].

Home-based and hybrid cardiac rehabilitation strategies are intriguing alternatives to center-based care due to these issues. Hybrid CR combines in-clinic supervision with home-based training, whereas home-based CR (HBCR) includes patients exercising and changing their lifestyle at home with remote coaching. Telemedicine and mobile health have made such approaches easier [4].

Remote CR programs can monitor patients in real time and offer feedback outside the hospital via phone or internet connectivity, smartphone apps, and wearable sensors [10,11]. These unique techniques attempt to preserve traditional CR effectiveness while overcoming logistical constraints to reach additional patients. Multiple research and evaluations suggest that telehealth-mediated CR is comparable to center-based programs in suitable individuals. For example, Huang et al. (2015) reported that tele-rehabilitation after cardiac events obtained results equivalent to supervised on-site rehab in exercise capacity and risk factor control [12]. Recent data indicate that home-based CR with wearable monitoring can be as effective as hospital-based rehab in improving fitness and risk profiles for patients with coronary disease or heart failure [6,13]. The use of wearable devices (e.g., smartwatches or activity trackers) in particular has been related with greater daily physical activity, improved adherence, and equal benefits in peak oxygen uptake and quality of life compared to standard care; such technology-enabled models gained further traction during the COVID-19 pandemic, which forced many CR programs to adopt remote protocols [3,6].

Building on this global trend, our institution implemented a 14-week hybrid cardiac rehabilitation program for post-MI (STEMI/NSTEMI with very high risk) patients with heart failure (NYHA class II), many of whom had multiple cardiac comorbidities.

The primary aim of this original research was to assess the efficacy of a technology-assisted hybrid cardiac rehabilitation program in post-MI patients with heart failure by analyzing the pre-rehabilitation data with post-hybrid rehabilitation data. We especially intend to show that extending CR outside the hospital context via a telemedicine platform generates considerably superior increases in exercise capacity, cardiovascular health indicators, and patient well-being than the confined conventional strategy. The research is innovative in many respects: (1) It focuses on a heart failure cohort (NYHA II) in the post-infarction phase, a population that is generally under-represented in rehabilitation studies. (2) It uses a cutting-edge wristwatch program (Mobile) to deliver and monitor exercise, constituting one of the first studies in our area to apply continuous remote vital sign monitoring and feedback in CR. (3) We systematically monitor a broad variety of patient outcomes (from fitness measurements to body composition and daily activities), offering a holistic perspective of how hybrid CR improves patient health.

## 2. Materials and Methods

### 2.1. Study Design

Patients were enrolled after undergoing complete revascularization, including both the culprit lesions responsible for the myocardial infarction and residual significant stenoses. The program was structured in two phases: an initial phase of in-hospital rehabilitation for 2 weeks, followed by a home-based phase of 12 weeks which was outpatient phase. In the initial phase, patients either underwent supervised exercise training as inpatients or daily outpatient sessions (5 days per week). This phase of rehabilitation was conducted in the Department of Medical Rehabilitation at Bagdasar-Arseni Hospital in Bucharest. Each patient’s regimen was individualized according to their discharge exercise EKG test results and clinical status, following standard CR protocols for post-MI/heart failure patients. Exercise intensity (target heart rate zones) and modalities were prescribed based on functional capacity and symptom tolerance under medical supervision. This phase served to stabilize the patient, provide education, and ensure they can exercise safely, while familiarizing them with the rehabilitation routine. For the subsequent 12-week home-based follow-up, patients transitioned to a remote rehabilitation platform, the Mobile application, combined with a Samsung smartwatch. This digital health solution was developed to monitor, guide, and personalize exercise training outside the hospital. Each patient was provided with a Samsung smartwatch from Bucharest, Romania synced to the Mobile smartphone app which was built with the React Native language and web-interface Drupal for the medical staff to see the data. The Mobile app provided daily reminders for exercise sessions and medication times, along with educational tips. A secure in-app chat allowed patients to directly communicate with the rehabilitation team to report symptoms, ask questions, or receive encouragement.

#### 2.1.1. Study Population and Inclusion Criteria

We prospectively enrolled 66 consecutive patients, 49 males (74.24%) and 17 females (25.8%), with a mean ± SD age of 54.24 ± 10.09 years, into our hybrid cardiac rehabilitation program at the Department of Cardiology at Bagdasar-Arseni Hospital in collaboration with Carol Davila University, Bucharest, Romania, starting in 2024. All participants were adults (≥18 years) under the care of the cardiology service and carried a diagnosis of heart failure, classified as New York Heart Association (NYHA) class II on the basis of clinical evaluation and supporting investigations (echocardiography and natriuretic peptide levels). Eligibility further required a recent history of myocardial infarction (within the preceding three months), possession of a compatible smartphone, and the cognitive and physical capacity to engage both in supervised exercise and remote monitoring via our proprietary Mobile application paired to a smartwatch. Patients were excluded if they declined participation, were unable to provide informed consent, had advanced heart failure (NYHA class III–IV), significant valvular heart disease (e.g., severe aortic stenosis or mitral regurgitation), end-stage renal impairment (estimated glomerular filtration rate ≤ 30 mL/min/1.73 m^2^), or any neurological or musculoskeletal condition precluding safe engagement in moderate-intensity exercise.

#### 2.1.2. Ethical Approval and Informed Consent

This research was done in compliance with the Romanian Constitutional Law 206/2004 (updated 2021), the Declaration of Helsinki, and European Union standards on professional ethics. Ethical permission was given by the Ethics Committee of Scientific Research at Carol Davila University of Medicine and Pharmacy, Bucharest (33193/02.11.2023). Written informed permission was acquired from all subjects before enrollment. The permission form specifically explicitly guaranteed patient anonymity, confidentiality, and the right to withdraw without affecting medical care. Data collection and management complied with institutional norms for personal data protection.

#### 2.1.3. Rehabilitation Program Design

Our hybrid cardiac rehabilitation model spanned fourteen weeks and combined two distinct but inter-related phases, designed to optimize both supervised and remote exercise training. During the first two weeks, patients attended five supervised sessions per week at our hospital’s rehabilitation unit. These sessions were conducted either as inpatients or on an outpatient basis, depending on clinical stability and logistical considerations. A standardized exercise protocol was applied, with each session individualized according to the results of a discharge exercise stress test (3 min step protocol—cycloergometer) performed after the acute myocardial infarction. Exercise intensity was prescribed using target heart rate reserve (HRR) zones and the Borg Rating of Perceived Exertion scale; continuous electrocardiographic and blood pressure monitoring ensured patient safety throughout each session. All participants received standard guideline-directed post-myocardial infarction medical therapy. During the initial in-hospital phase, cardiovascular medications were adjusted only when clinically indicated by the treating cardiologist, and no protocol-mandated therapeutic modification was introduced as part of the rehabilitation intervention itself.

Following this initial in-hospital phase, patients transitioned to a twelve-week home-based tele-rehabilitation program delivered via the Mobile application. Each patient received a smartwatch at discharge, which seamlessly synchronized with their personal smartphone and the Mobile platform. The smartwatch has obtained CE-mark certification in the European Economic Area as a medical device, specifically Class IIa under EU MDR. In addition, the ECG on compatible Watch models has received FDA De Novo authorization for over-the-counter use in adults ≥ 22 years. This digital ecosystem sent daily reminders, guided daily exercise sessions through personalized demonstrations, tracked real-time heart rate on a second-by-second basis, and recorded resting heart rate at ten-minute intervals. In addition, patients received automated reminders for medication adherence. Blood pressure values were manually entered by patients using standardized equipment and recorded in the mobile application, alongside laboratory results and other medical investigations. All this information was accessible in real time to the healthcare team, enabling timely and personalized recommendations. When a patient’s heart rate fell outside the prescribed target HRR zone, the app generated automated alerts advising them either to increase intensity, reduce effort, or pause the session. If the recorded values exceeded or dropped below predefined safety thresholds, the assigned caregiver was also notified in real time, enabling direct contact with the patient for immediate assessment and guidance. In addition to cardiovascular monitoring, the smartwatch utilized bioimpedance technology to measure body composition parameters including total body weight, fat mass, total body water, and skeletal muscle mass each morning. Patients also wore the device throughout daily activities, enabling continuous step-count tracking and passive activity logging.

The Mobile app featured an integrated symptom-reporting function; whenever patients experienced chest discomfort, palpitations, or dyspnea, they could transmit a single-lead ECG recording directly through the app for automatic preliminary analysis; any abnormal tracings generated by the algorithm were flagged for rapid review by the medical team. Secure in-app messaging allowed patients to consult their rehabilitation specialists in real time, ask questions about exercise tolerance or medication adjustments, and receive tailored reminders and motivational messages to foster adherence.

#### 2.1.4. Data Collection and Management

Baseline data were captured at the conclusion of the two-week supervised phase. These included exercise EKG stress-test parameters (total exercise duration, peak watts, and watts per kilogram), resting hemodynamics (blood pressure and heart rate), and body composition measures. Throughout the subsequent twelve weeks, the Mobile platform continuously collected daily step counts, resting heart rate readings, blood pressure values (measured by patients using an automated blood pressure monitoring device and then inputted in the app and transmitted via the app), and any symptom-driven ECG uploads. At the end of week fourteen, follow-up measurements mirrored the baseline assessments; for example, a repeat exercise EKG stress test provided updated exercise capacity metrics, and morning bioimpedance readings yielded post-rehabilitation body composition data. All device-derived and clinical data were stored in a secure database, with access restricted to the study team.

#### 2.1.5. Sample Size

A formal sample-size calculation was performed in G*Power version 3.1 (Heinrich-Heine-Universität Düsseldorf, Düsseldorf, Germany) [14] to verify that our cohort would be sufficiently powered to detect meaningful within-subject improvements in both exercise capacity and body composition metrics. We specified a two-tailed paired-samples *t*-test, an alpha threshold of 0.05, and a desired power of 0.80. Drawing on prior tele-rehabilitation studies in post-myocardial infarction patients, we assumed a moderate standardized effect size (Cohen’s d) of 0.50 for key endpoints (e.g., peak exercise watts and body fat percentage). Under these parameters, G*Power calculated that a minimum of 54 participants would be required to achieve 80% power. Because our analysis ultimately included 66 patients who completed both the in-hospital and home-based phases of the hybrid rehabilitation, the achieved power rose to approximately 98%, conferring a robust ability to detect moderate effects. Enrolling 66 subjects therefore not only surpassed the a priori requirement but also enhanced estimate precision, lowered the standard error, and further guarded against type II error in our comparisons.

#### 2.1.6. Statistical Analysis

All analyses were conducted in IBM SPSS Statistics v28.0. Continuous variables were first screened for normality using the Shapiro–Wilk test to guide the choice of parametric versus nonparametric methods. Descriptive statistics are presented as mean ± SD for continuous measures and counts (percentages) for categorical variables. Within-subject changes from baseline to week 14 formed the primary endpoints. Maximal MET change scores violated normality and were analyzed with a Wilcoxon signed-rank test. Variables meeting normality such as systolic and diastolic blood pressure and skeletal muscle mass were compared using paired-samples *t*-tests, with mean differences reported alongside 95% confidence intervals (CIs), t-statistics, two-tailed *p*-values, and Cohen’s d. Additional non-normal outcomes (NYHA class, body fat mass, daily steps, and total body water) were similarly evaluated with Wilcoxon signed-rank tests, yielding standardized Z-values, exact *p*-values, and effect sizes. All tests were two-tailed with α = 0.05.

## 3. Results

We included 66 patients in this single-center study; there were 49 males (74.24%) and 17 females (25.8%), and ages spanned from 30 to 80 years, with a mean value of 54.24 ± 10.09 years and no significant departures from normality (skewness −0.347 ± 0.295). Table 1 summarizes the baseline characteristics, comorbidities, and clinical outcomes (n = 66)

Among the 66 participants, gender was coded as 1 = male and 2 = female, yielding a mean of 1.26 ± 0.44 and a skewness of 1.135 ± 0.295. A total of 49 patients (74.2%) were male and 17 (25.8%) were female.

### 3.1. Comorbidities

Regarding the comorbidities of the selected patients, smoking prevalence was 60.6% (mean 0.61 ± 0.49; skewness −0.444 ± 0.295). Hypertension affected 69.7% (mean 0.70 ± 0.46; skewness −0.877 ± 0.295), and diabetes mellitus 24.2% (mean 0.24 ± 0.43; skewness 1.230 ± 0.295). All patients had had a prior myocardial infarction (mean 1.00 ± 0.00).

Extrasystolic arrhythmia was present in 37.9% (mean 0.38 ± 0.49; skewness 0.511 ± 0.295), while dyslipidemia was noted in 90.9% (mean 0.91 ± 0.29; skewness −2.913 ± 0.295).

### 3.2. New York Heart Association (NYHA) Class

Utilizing the comorbidity profile of the patients in the study, we next looked at how their functional status changed before and after the 14-week hybrid rehabilitation utilizing the New York Heart Association (NYHA) class. Forty patients (61%) stayed in the same NYHA class (ties), while twenty-six (39%) moved down a class, showing improvement. A Wilcoxon signed-rank test showed a very significant median decrease in NYHA class (standardized Z = −5.099; asymptotic *p* < 0.001), which prompted us to reject the null hypothesis that there was no change. These findings show that our intervention made a real difference in the clinical functional class of the patients who took part in the hybrid rehab program.

### 3.3. Weight of Patients

We next looked at changes in body weight across the 14-week intervention, which are shown in Table 1, based on the improvements we saw in NYHA class. The average weight went down from 89.00 ± 15.67 kg at the start to 87.74 ± 15.52 kg at the follow-up, which is a mean paired difference of 1.27 ± 2.51 kg (95% CI 0.65–1.88 kg). This decrease was highly significant (t = 4.089; df = 65; *p* < 0.001) and was equal to a moderate standardized effect (Cohen’s d = 0.50; 95% CI 0.25–0.76; Hedges’ g = 0.49; 95% CI 0.24–0.75), which means that the people in the study lost weight.

### 3.4. Blood Pressure Changes

Moreover, following the 14-week hybrid rehabilitation, systolic blood pressure fell by an average of 5.1 mmHg (from 125.3 to 120.2 mmHg; *p* < 0.001), and diastolic pressure decreased by 2.2 mmHg (from 76.4 to 74.2 mmHg; *p* = 0.002). These changes corresponded to moderate (Cohen’s d = 0.62) and small-to-moderate (d = 0.39) standardized effects, respectively, indicating that the combined in-hospital and home-based program produced statistically and clinically meaningful improvements in the hemodynamics of the patients.

### 3.5. Maximal Metabolic Equivalents (METS)

Furthermore, we performed paired evaluations of maximum metabolic equivalents (METS) before and after the hybrid rehabilitation program. Maximal METS is the greatest level of exercise intensity a patient may accomplish. It is thought of as how many times harder an individual’s body is working during peak exercise compared with when he or she is resting. It is a simple approach to measure the total cardiovascular fitness and anticipate health effects. Higher values represent better fitness and lower risk. METS were estimated based on the maximum workload effectively reached by each patient during the symptom-limited EKG exercise stress test performed on a cycloergometer. The estimation used the formula METS = [(Workload in WATSS × 10.8/body weight in kg) + 3.5]/3.5. The distribution of difference scores was analyzed using the Wilcoxon signed-rank test. Sixty subjects indicated positive increases in METS, and six showed no change. The week 14 reading showed an approximately 25.7% improvement in METS compared to the week 1 reading; patients gained 1.685 METS compared to the first reading. The test gave a two-tailed *p* < 0.001, prompting us to reject the null hypothesis of no median change. These findings demonstrate a very substantial increase in exercise ability after the 14-week intervention. These results are presented in Table 2.

Furthermore, changes in resting heart rate over the 14-week hybrid rehabilitation program were observed. Resting heart rate declined significantly from week 1 to week 14 (Wilcoxon signed-rank Z = −3.116; *p* = 0.002). The resting heart rate at week 1 was 75.68 ± 7.38, and the heart rate in the final week was 72.80 ± 6.67, indicating improved autonomic regulation at rest shown in Table 3a. Meanwhile, peak heart rate attained during exercise stress testing decreased modestly from a mean of 134.73 ± 20.34 bpm at baseline to 131.58 ± 17.53 bpm at follow-up as shown in Table 3b. However, the 3.15 ± 15.29 bpm reduction did not reach statistical significance (95% CI −0.61 to 6.91 bpm; t (65) = 1.674; *p* = 0.099), corresponding to a small effect size (Cohen’s d = 0.21; 95% CI −0.04 to 0.45). This is shown in Table 3a,b.

Next, we evaluated changes in exercise efficiency as measured by watts per kilogram (W/kg) during maximal stress testing. At baseline, participants achieved a mean of 1.405 ± 0.385 W/kg, which increased to 1.784 ± 0.941 W/kg at week 14, which indicated an improved power output relative to body mass. A related-samples Wilcoxon signed-rank test was performed to accommodate the skewed distribution of the change scores. The test yielded a standardized statistic of Z = 7.010 (T = 2145.000; SE = 152.988), with a two-tailed *p* < 0.001, leading us to reject the null hypothesis of no median change. These obtained results demonstrate that our 14-week hybrid rehabilitation program produced a substantial and statistically robust enhancement in relative exercise capacity. The shift in W/kg captures both increased absolute power generation and favorable body composition changes, underscoring the multifaceted benefits of integrating in-hospital supervised training with a remotely monitored, app-guided home phase. The findings are presented in Table 4.

The hybrid rehabilitation program produced significant and clinically relevant changes not only in exercise capacity and cardiovascular parameters, but also in body composition and physical activity levels. Detailed analyses of body fat mass, skeletal muscle mass, daily step counts, and total body water are presented subsequently.

### 3.6. Body Fat Mass

At baseline week 1, mean body fat mass was 27.21 ± 8.98 kg, which declined to 25.37 ± 9.24 kg at week 14. A related-samples Wilcoxon signed-rank test yielded a significant result with (*p* < 0.001). Summarized in Table 5.

The muscle mass of the patients rose from an average of 33.42 ± 4.45 kg at the start of the rehabilitation program to 34.40 ± 4.49 kg at follow-up, a gain of 0.98 kg (95% CI 0.54–1.42 kg). This improvement was statistically robust (*p* < 0.001; CI (−0.80 to −0.29)), indicating that the increase was very unlikely to be due to chance. This finding reflected a meaningful, moderate-strength change in participants’ body composition. This is shown in Table 6.

A daily activity such as walking was highly recommended to the patients and reminders were sent at specific times too via the Mobile app. Patients felt motivated to walk during the rehabilitation duration. Average daily steps rose markedly from 5549.7 ± 2025.5 at week 1 to 7267.3 ± 2500.0 at week 14. As summarized in Table 7.

Finally, total body water of the patients was calculated and monitored too at the start of the rehabilitation program and at the end at week 14. The total body water content increased from 45.50 ± 5.41 kg to 46.10 ± 5.72 kg (*p* < 0.001). It is shown in Table 8.

Our presented findings underscore that the 14-week hybrid program not only improved functional capacity and hemodynamics but also favorably modified body composition and physical activity levels, supporting its multifaceted efficacy in post-MI cardiac rehabilitation.

## 4. Discussion

Several cardiovascular health categories were analyzed in this single-center study and numerous outcomes improved in this 14-week hybrid cardiac rehabilitation (CR) program for post-myocardial infarction NYHA class II heart failure patients. Stress peak METS and walk test lengths rose significantly, indicating enhanced exercise capacity. In addition to weight and body composition improvements, resting blood pressure and heart rate decreased. Many patients improved from NYHA II to class I, showing symptom and exercise tolerance improvements. These findings show that a hybrid CR model combining supervised in-clinic exercise sessions with home-based training can safely improve exercise performance, modifiable risk factors, heart failure functional class, and fitness in this high-risk population. The clinical improvements observed in our study, including enhanced exercise capacity, improved NYHA functional class, and favorable hemodynamic changes, are increasingly understood to be driven not only by peripheral conditioning but also by complex molecular and epigenetic adaptations induced by cardiac rehabilitation. Recent evidence by Visco et al. highlights that exercise-based CR modulates gene expression through epigenetic mechanisms, including DNA methylation, histone modification, and regulation of non-coding RNAs [15].

At the cellular level, these adaptations promote cardiomyocyte survival, reduce apoptosis, and enhance myocardial contractility and regeneration. For instance, exercise has been shown to upregulate cardioprotective microRNAs such as miR-126 and miR-222, which are associated with angiogenesis, anti-apoptotic signaling, and improved cardiac repair following myocardial injury. Additionally, modulation of long non-coding RNAs (e.g., MALAT-1 and MIAT) contributes to reduced fibrosis and improved autophagy, further supporting myocardial recovery.

Moreover, exercise-induced epigenetic changes also influence inflammatory pathways and metabolic efficiency. Increased DNA methylation of pro-inflammatory mediators and histone modifications regulating key metabolic genes have been associated with improved endothelial function and myocardial energy utilization. These molecular adaptations may partly explain the significant improvements in functional capacity (e.g., MET increase) and reduction in resting heart rate observed in our cohort. Our results align closely with a growing body of literature supporting the efficacy of hybrid and non-traditional CR models. Evidence has long shown that standard exercise-based CR improves patients’ aerobic capacity, muscular strength, and quality of life, while conferring prognostic benefits such as reduced mortality and hospitalizations [16,17,18]. For instance, participation in CR has been associated with an approximately 26% reduction in cardiovascular mortality and significant drops in recurrent myocardial infarction and hospital readmission rates [16,19]. An observational study conducted by Suaya et al. strongly advocates for CR and found a 3-year mortality reduction of around 34% in CR patients versus 21% in non-participants [20], underscoring the life-saving potential of CR. These benefits have been documented even in older patients; classic studies by Lavie and Milani and others reported that elderly cardiac patients achieve improvements in exercise capacity and risk profiles comparable to their younger counterparts [21]. Our finding of improved METS and symptom class in a cohort with a mean age in the mid-60s is consistent with those observations.

Center-based supervision and home-based instruction in hybrid CR appear to retain the benefits of classic CR while resolving access hurdles [17,22]. Recent studies and reviews show that hybrid or home-based CR increases exercise capacity just as well as center-based programs [23,24]. In the FIT@Home randomized controlled trial by Kraal et al. (2014), 12 weeks of home-based, telemonitored exercise resulted in a 14% increase in peak VO_2_, comparable to clinic-based CR’s 10% improvement, and there was no difference in training adherence [24]. Carefully organized remote or hybrid exercise can match hospital-based regimens in efficacy, as shown by our program’s 25.7% MET increase and improved QoL measures. Yuen et al. (2023) reported that a longer hybrid CR (8-week program with weekly on-site sessions and at-home exercise) reached peak MET levels comparable to a 5-week, fully supervised program [25]. Yuen et al. [25] reported a 1.6 MET increase, which matches our program’s 1.685 MET rise. Heindl et al.’s 2022 [23] review of hybrid CR programs found that hybrid models improve exercise capacity, risk factor control like lipid profiles and blood pressure, and health-related quality of life just as well as center-based CR in coronary artery disease patients. Our post-MI heart failure sample showed significant decreases in resting blood pressure and weight, confirming these short-term advantages. Weight reduction was moderate, although BMI and body fat decreases during 3-month CR without dietary modification are often minor [17]. The large functional gains confirm that hybrid CR is a safe and effective cardiopulmonary fitness regimen for stable NYHA II patients, supporting the idea that exercise training is well-tolerated even in chronic heart failure.

The ability to offer flexibility is one reason hybrid CR is used to promote patient uptake and adherence. Our study found that most patients completed >80% of the 14-week regimen, possibly due to the home-based component minimizing travel stress. Recent research shows that hybrid CR improves program completion and patient satisfaction compared to standard models. A meta-analysis by Anderson et al. reported no differences in mortality, morbidities, or exercise ability between home-based and center-based CR, while home-based methods had slightly higher completion rates (~4% higher; RR 1.04) [26]. The multicenter Hybrid CR Trial (HYCARET) in Chile found that hybrid program patients had comparable clinical results to regular CR patients. Hybrid CR may improve supervised exercise adherence over time [27].

Hybrid models may do this by reducing distance, travel time, work/family conflicts, and facility access, which disproportionately affect women, elderly people, and rural patients. Home-based monitoring with smart device reminders and automated parameter monitoring frequently overcomes these limitations. Hybrid CR can improve CR utilization and availability to underserved groups by bringing parts of the program home [22]. Our successful engagement of post-MI heart failure patients (a group often under-referred to CR) highlights how hybrid delivery may advance health equity in secondary prevention. Furthermore, resource-constrained settings stand to gain from hybrid CR’s efficiency: with fewer in-person sessions, programs can accommodate more patients and reduce costs without sacrificing outcomes [18,23]. This has been corroborated by economic analyses like the FIT@Home study, which found home-based CR more cost-effective than center-based CR due to similar clinical results achieved at lower expense [24].

Our cohort’s subjective and objective reporting of symptom alleviation, exercise self-efficacy, and well-being improvements are consistent with hybrid CR studies. Exercise tolerance improves everyday functioning and health-related quality of life specifically in heart failure, and exercise-based rehabilitation has been shown to improve fatigue, emotional distress, and overall health status. The HF-ACTION trial, while focused on center-based exercise, demonstrated a significant improvement in KCCQ quality-of-life score, with a value 5 points higher than controls at 3 months in the exercise arm [28]. Our patients’ subjective perceptions of enhanced stamina and confidence in executing activities are aligned with the aforementioned data. Notably, NYHA class improved in a considerable proportion of patients; those who were class II at baseline reported very minor or no symptoms (class I) following training, showing major functional progress. Although NYHA class was not a formal endpoint in most hybrid CR research, comparable changes have been reported indirectly by better peak VO_2_ and 6 min walk lengths in exercise trials for heart failure. For instance, a tele-rehab study in stable chronic HF with a mean age of 67 ± 12 years demonstrated that 6 weeks of home-based exercise was non-inferior to center-based rehab in increasing 6 min walk distance, resulting in equivalent functional classes post-intervention. Likewise, our hybrid program’s aerobic activity and exercise likely led to superior skeletal muscle function and endurance, which are significant predictors of functional class. Beyond the physical measurements, the psychological and social support inherent in CR (even when offered remotely) may increase patients’ confidence and morale, further raising perceived quality of life.

While the overall evidence strongly favors CR (in any format) for secondary prevention, not all trials of exercise-based cardiac rehabilitation have demonstrated outcome improvements, especially in contemporary patient populations receiving optimal medical therapy. A notable example is the RAMIT trial in the UK, a large pragmatic RCT of post-MI patients that found no significant differences in all-cause mortality or recurrent myocardial infarction between those referred to CR and those receiving usual care [29]. Furthermore, RAMIT reported no improvement in psychosocial well-being or quality of life, and even observed lower self-reported physical activity at 1 year in the CR group. These counterintuitive results which contrast with our findings and most of the literature could be attributed to trial limitations such as suboptimal CR program adherence, an outdated rehab model, and insufficient power after early trial termination and less innovative technological solutions. Another area of debate is the long-term sustainability of hybrid or home-based CR benefits. Pratesi et al. (2019) [18] investigated an extended hybrid approach in elderly cardiac patients where a standard 4-week outpatient CR was followed by 6 months of home exercise with monthly reinforcement. They found that, although the initial CR led to significant gains in VO_2_ peak, 6 min walk distance, and strength (similar to our 14-week results), the group receiving the additional home-based phase had no further functional advantages at 1-year follow-up compared to controls who received usual post-CR care [18]. The add-on home exercise did not yield incremental long-term improvements beyond the early rehab gains. Our study was not powered to assess long-term clinical events but the weight of evidence suggests that hybrid CR should confer prognostic advantages similar to traditional CR provided that patients remain engaged and the program is well integrated with their ongoing care.

## 5. Limitations

Like all studies, our study has some limitations too which must be addressed. Our study’s main limitations stem from its single-center, uncontrolled pre–post design and modest sample size (n = 66), and participants were predominantly male which precludes causal inferences and limits generalizability to broader post-MI or heart failure populations. This study design introduces selection bias as well due to its single-center nature. In the absence of a contemporaneous control group or randomization, the observed improvements may reflect practice-related learning effects or behavioral changes resulting from participants’ awareness of being observed (Hawthorne bias). Reliance on patient-reported home blood pressure and app-recorded activity may introduce measurement error, whereas lack of dietary or medication adherence monitoring confounds interpretation of weight and hemodynamic changes. The absence of a control group and the pre–post (within-subject) design limit causal inference, as the observed improvements may partly reflect behavioral changes, regression to the mean, or the Hawthorne effect. The 14-week follow-up captures only short-term gains, leaving long-term sustainability and hard clinical outcomes unaddressed. It remains to be seen if the gains in exercise capacity translate into sustained physical activity habits and reduced mortality or rehospitalization over years, which is a question that future research should address. Moreover, as much as we tried to control the confounders, some confounders such as medication optimization, dietary changes, or variations in baseline physical activity were not controlled. Lastly, because this was a real-world clinical study, minor adjustments in cardiovascular medical therapy during the early in-hospital phase could not be fully excluded and may have contributed to some of the observed improvements, particularly in hemodynamic parameters.

## 6. Future Implications

Future implementation of hybrid cardiac rehabilitation should prioritize multicenter, randomized trials with larger sample sizes to confirm efficacy and generalizability. Based on the validated protocol developed and applied in the present study, initial collaborative efforts have already been established with multiple cardiology departments across the country. These partnerships aim to replicate the intervention model in varied clinical settings and patient cohorts. Moreover, preliminary discussions are underway to facilitate the international adoption of this framework, with the long-term goal of integrating hybrid cardiac rehabilitation into broader, cross-border preventive cardiology strategies. Integrating a contemporaneous control arm will help isolate the true intervention effect and minimize biases. Extending follow-up beyond 14 weeks is essential to assess the sustainability of functional gains and their impact on hard endpoints such as rehospitalization and mortality. Moreover, cost-effectiveness analyses and patient-reported outcome measures will inform real-world adoption, while stratified analyses can identify which subgroups segregated by age, comorbidity burden, or baseline fitness derive the greatest benefit. Finally, novel strategies such as mobile health apps and activity trackers might be leveraged in future hybrid CR to promote long-term exercise maintenance.

## 7. Conclusions

This 14-week hybrid cardiac rehabilitation program for post-MI patients with NYHA class II heart failure achieved meaningful gains: mean METS rose by approximately 1.7 at the end of the study; resting heart rate and blood pressure improved; body weight and composition shifted favorably; and NYHA class declined at adherence levels comparable to center-based models. Our results align with existing CR evidence, demonstrating that combining supervised sessions with home-based monitoring can deliver durable fitness and symptom benefits while broadening access. However, we emphasize that these results are hypothesis-based and future work such as randomized controlled trials should confirm long-term outcomes and refine hybrid delivery for diverse patient groups. Nonetheless, it is a feasible and potentially effective alternative to conventional programs in resource limited settings.

## Figures and Tables

**Table 1:** Baseline characteristics, comorbidities, and clinical outcomes (n = 66).

Section	Parameter	Value
Demographics	Age (years)	54.24 ± 10.09
	Range	30–80
	Skewness (SE)	−0.347 (0.295)
Gender Distribution	Male	49 (74.2%)
	Female	17 (25.8%)
	Total	66 (100%)
Comorbidities	Smoker	40 (60.6%)
	Hypertensive	46 (69.7%)
	Diabetic	16 (24.2%)
	Prior myocardial infarction	66 (100.0%)
	Extrasystolic arrhythmia	25 (37.9%)
	Dyslipidemia	60 (90.9%)
NYHA Class Change	Total N	66
	Number of ties	40
	Test statistic (T)	0.000
	Standardized Z	−5.099
	*p*-value	<0.001
Weight Change	Initial weight (kg)	1 ± 15.67
	Final weight (kg)	87.74 ± 15.52
	Mean difference (kg)	1.27 ± 2.51
	95% CI	0.65–1.88
	t-statistic	4.089
	*p*-value	<0.001
	Cohen’s d	0.50 (0.25–0.76)
	Hedges’ g	0.49 (0.24–0.75)
Blood Pressure Change	Initial SBP (mmHg)	125.32 ± 12.61
	Final SBP (mmHg)	120.20 ± 9.35
	Mean Δ SBP	5.12 ± 8.28
	*p*-value (SBP)	<0.001
	Cohen’s d (SBP)	0.62 (0.35–0.88)
	Initial DBP (mmHg)	76.44 ± 9.07
	Final DBP (mmHg)	74.23 ± 7.63
	Mean Δ DBP	2.21 ± 5.63
	*p*-value (DBP)	0.002
	Cohen’s d (DBP)	0.39 (0.14–0.64)

**Table 2:** Comparison of initial and last metabolic equivalents on EKG.

Variable	N	Minimum	Maximum	Mean	Standard Deviation	Positive Differences	Standard Error (SE)	Asymptotic *p* (2-Tailed)
Max Metabolic Equivalents on First EKG	66	4.10	9.80	6.573	1.2369	—	—	—
Max Metabolic Equivalents on Last EKG	66	4.90	65.00	8.258	7.2489	—	—	—
First vs. Last EKG Comparison	66	—	—	—	—	60	135.796	<0.001

**Table 3:** (**a**) Resting heart rate change; (**b**) changes in peak exercise and resting heart rate pre- and post-rehabilitation.

(**a**)	
**Parameter**	**Value**
Heart rate week 1	75.68 bpm
Heart rate final week	72.80 bpm
Standard error	116.016
Asymptotic *p* (two-tailed)	0.002
(**b**)	
**Parameter**	**Value**
Baseline mean ± SD (bpm)	134.73 ± 20.34
Follow-up mean ± SD (bpm)	131.58 ± 17.53
Mean difference ± SD (bpm)	3.15 ± 15.29
95% CI of difference (bpm)	−0.61 to 6.91
*p*-value (two-tailed)	0.099
Cohen’s d (95% CI)	0.21 (−0.04 to 0.45)

**Table 4:** Paired-samples statistics and effect sizes for weight/kg change.

Parameter	Value
Baseline W/kg, mean ± SD	1.405 ± 0.385
Follow-up W/kg, mean ± SD	1.784 ± 0.941
Asymptotic *p* (two-tailed)	<0.001

**Table 5:** Body fat mass pre- and post-rehabilitation.

Parameter	Value
Initial fat mass, mean ± SD (kg)	27.21 ± 8.98
Final fat mass, mean ± SD (kg)	25.37 ± 9.24
Asymptotic *p* (two-tailed)	<0.001

**Table 6:** Skeletal muscle mass pre- and post-rehabilitation program.

Parameter	Value
Initial muscle mass, mean ± SD (kg)	33.42 ± 4.45
Final muscle mass, mean ± SD (kg)	34.40 ± 4.49
Mean difference ± SD (kg)	0.98 ± 1.80
95% CI of diff. (kg)	0.54–1.42
*p* (two-tailed)	<0.001
Cohen’s d (95% CI)	–0.55 (−0.80 to −0.29)

**Table 7:** Steps taken by participants at week 1 versus week 14.

Parameter	Value
Week 1 steps, mean ± SD	5549.7 ± 2025.5
Week 14 steps, mean ± SD	7267.3 ± 2500.0
Asymptotic *p* (two-tailed)	<0.001

**Table 8:** Total body weight pre- and post-rehabilitation.

Parameter	Value
Initial TBW, mean ± SD (kg)	45.50 ± 5.41
Final TBW, mean ± SD (kg)	46.10 ± 5.72
Asymptotic *p* (two-tailed)	<0.001

## Data Availability

All data generated or analyzed during this study are included in this article. Further enquiries can be directed to the corresponding authors.
